# The Age-Dependent Contribution of Aortic Incident and Reflected Pressure Waves to Central Blood Pressure in African-Americans

**DOI:** 10.4061/2011/585703

**Published:** 2011-08-24

**Authors:** Haroon Kamran, Jason M. Lazar, Rinkesh Patel, IIir Maraj, Heather Berman, Louis Salciccioli

**Affiliations:** Division of Cardiovascular Medicine, State University of New York Downstate Medical Center, Brooklyn, NY 11203-2098, USA

## Abstract

Aging is associated with increased central aortic systolic pressure (CSP) and pulse pressure which are predictive of cardiovascular events. Mechanisms implicated for higher central pressures include a higher forward incident pressure wave (P1), higher augmented pressure (AP), and shorter reflected wave round trip travel time (Tr). African-Americans (AA) have more frequent and deleterious blood pressure elevation. Using applanation tonometry, we studied the association of age and CSP with P1 and AP in 900 AA subjects. Data showed that in subjects ≤50 years old, CSP was mediated by AP but not P1 or Tr, whereas in those >50, CSP was mediated by both AP and P1 and to a lesser extent by Tr. Predictive models were significant (*R* ^*2*^ = 0.97) for both age groups. In conclusion, wave reflection is the primary determinant of CSP in younger AA, while in older subjects, CSP is mediated by both the magnitude and timing of wave reflection as well as aortic impedance.

## 1. Introduction

Vascular aging and remodeling predominantly affects the large elastic arteries, with the unfavorable consequence of increased aortic stiffness and higher central systolic (CSP) and pulse pressure (PP) [1]. In recent years, it has been demonstrated that central pressures are more closely related to cardiovascular outcomes as compared to peripheral pressures [2]. Pathological changes within the arterial system lead to hemodynamic alterations that are reflected in the aortic waveform. The growing use of applanation tonometry has rejuvenated interest in the mechanisms of blood pressure elevation. Several mechanisms have been proposed for the higher central pressures observed with aging. There may be an increased forward incident pressure wave (P1), a higher augmented pressure (AP) and augmentation index (AI), and a shorter round trip travel time of the reflected wave (Tr) [3]. Aortic characteristic impedance also increases with age, thereby increasing P1. Greater AP due to increased wave reflection is the conventional explanation of why CSP increases with age, with age-related increases in aortic stiffness shortening Tr and causing the reflected wave to sum on the incident wave during systole [4–8]. Other investigators have found P1 to be the more important determinant of central pressure [9, 10]. A recent study in a large cohort of normal subjects found the contribution of AP and P1 to the CSP to vary between those younger and older than 60 years [11]. Thus, the reasons for higher CSP and PP with advancing age have not been fully clarified.

African-Americans (AA) have a high prevalence of hypertension, and greater propensity for end-organ damage including left ventricular hypertrophy [12]. They have also been reported to have higher aortic stiffness [13]. The age-related contributions of the incident and reflected waves to CSP in this group of individuals have not been well studied. Accordingly, we studied the age-related contribution of the incident and reflected waves to CSP and PP in a large cohort of AA subjects with and without cardiovascular disease.

For our analysis, we divided the cohort into 2 age groups, ≤50 years and those >50 years old. This is based on prior studies demonstrating AI to increase until age 60 and then level off, and that aortic stiffness measured by pulse wave velocity (PWV) increases predominantly in those >50 years old [14, 15]. Additionally, in this current study, we found that age correlated with P1 only in those subjects >50 years old.

## 2. Materials and Methods

The hospital institutional review board approved this study, and all patients provided written consent. We prospectively studied 900 AA subjects (age 58 ± 17 years) without known congestive heart failure. Inclusion criteria were age ≥18 years, adequate radial and carotid pulses to obtain the applanation tonometry study, and sinus rhythm. Peripheral systolic (S) and diastolic (D) blood pressures (BP) were measured using an automated sphygmomanometer device (Omron HEM-780). Pulse pressure (PP) was defined as the difference between SBP and DBP. Brachial artery mean arterial pressure (MAP) was calculated as diastolic pressure +1/3 PP. The central aortic waveform was derived from the radial artery waveform with the use of applanation tonometry (Sphygmocor, Atcor Medical) and a validated transfer function [16, 17]. The reflected wave pressure (AP) amplitude was defined as the difference between peak SBP and pressure at the inflection point of the aortic waveform [18]. The AI was defined as the proportional increase in SBP due to the reflected wave and was expressed as a percentage of the PP, and since heart rate dependent was also corrected for heart rate of 75 bpm (AI75) [18]. Tr is defined as the time from the initial upstroke of the pressure wave to the incident pressure P1. P1 is defined as the pressure difference between the inflection point and the diastolic pressure. The reflected wave systolic duration (ΔTr) is determined from the inflection point to the incisura [18]. The ejection duration is the time from the initial pressure upstroke to the incisura. These were obtained from the derived aortic waveform as previously described [18].

Clinical characteristics evaluated included age, gender, medication use, body mass index, history of hyperlipidemia, diabetes mellitus, hypertension, chronic renal insufficiency, known coronary artery disease defined as any degree of coronary disease as per patient history or chart review, previous stroke, and smoking status (current cigarette use). Hyperlipidemia, hypertension, chronic renal insufficiency, and diabetes mellitus were defined either as self-reported, documented diagnosis obtained from chart review, or current treatment with medication. As noted previously, for the statistical analysis of the age-related contributions of the waveform characteristics to central pressures, we divided our cohort into those ≤50 years and those >50 years old.

## 3. Statistics

Data is presented as mean ± standard deviation (SD). Differences between younger (≤50 years) and older subjects (>50 years) were assessed using independent sample *T* test for continuous variables and with Fischer Exact or Chi-square test for dichotomous variables. The contribution of age with and without the mediation of AP and P1 to the CSBP and CPP was assessed using both mediation analysis and linear regression adjusted for the potential cofounders of gender, height, weight, HR, MAP, and CV risk factors or known coronary disease using SPSS statistical analysis software (version 17). Mediation analysis was performed both with and without the inclusion of medication classes (beta-blocker agents, angiotensin converting enzymes inhibitors and angiotensin receptor blocking agents (ACE/ARB), and calcium channel blockers) to exclude any medication interaction. Mediation analysis was used on the premise that mediation exists when a predictor affects a dependent variable indirectly through at least 1 intervening variable, or mediator. With increasing age, there is increased AP and P1, and, due to these changes, CSP and PP rise; thus, the central pressure and age relationship may be mediated by AP and P1. Mediation analysis has been modified by Preacher and Hayes to include multiple mediators and covariates [19].

## 4. Results

**Figure 1:**
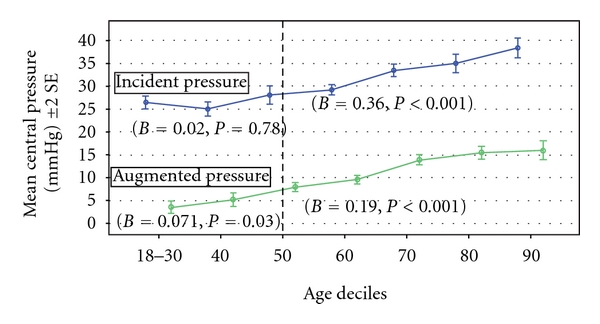
Augmented pressures and incident pressures in younger and older subjects by deciles of age.

Table 1 shows characteristics of the study subjects. Thirty-two percent of subjects were ≤50 years. Thirty seven percent were taking a beta-blocking agent, 34% an ACE/ARB, and 18% a calcium-blocking agent. On univariate analysis, there were significant correlations between AP (*r* = 0.55, *P* < .001) and P1 (*r* = 0.46, *P* < .001) with CSP before and after 50 years of age (*r* = 0.67, *P* < .001 and 0.69, *P* < .001, resp.). On adjusted multivariate linear regression, in subjects ≤50, age correlated with AP (B = 0.071, *P* = 0.03) but not with P1 (B = 0.02, *P* = 0.78) or Tr (B = −0.17, *P* = 0.13). After age 50, age correlated positively with both AP and P1 (B = 0.19, *P* < 0.001 and B = 0.36, *P* < 0.001, resp.) and inversely with Tr (B = −0.17, *P* = 0.007). Mediation analysis demonstrated in younger subjects that CSP was mediated by AP (B = 0.07, 95% CI = 0.01 − 0.14, *P* < 0.05), but not by P1 or Tr (B = −0.001, 95% CI = −0.036–0.027, *P* = NS and B = −0.008, 95% CI = −0.03–0.0001, *P* = NS, resp.). In subjects >50, the CSP was similarly mediated by AP and P1 (B = 0.16, 95% CI = 0.11–0.25, *P* < 0.05 and B = 0.17, 95% CI = 0.09–0.22, *P* < 0.05, resp.), and to a lesser extent by Tr (B = −0.01, 95% CI = −0.03– −0.002, *P* < 0.05). Mediation analysis adjusted models were highly significant with an *R* ^2^ = 0.97 in both younger and older subjects (*P* < 0.001 for both). When CPP was substituted for CSP, results were similar to those for CSP. In subjects ≤50 years of age, CPP was mediated only by AP (B = 0.09, 95% CI = 0.02–0.19, *P* < 0.05) and in those age >50 the relationship of age with CPP was similarly mediated by AP and P1 (B = 0.20, 95% CI = 0.15–0.34 and B = 0.29, 95% CI = 0.15–0.38, *P* < 0.05, resp.).  Figure 1 shows changes in AP and P1 by deciles of age.

**Figure 2:**
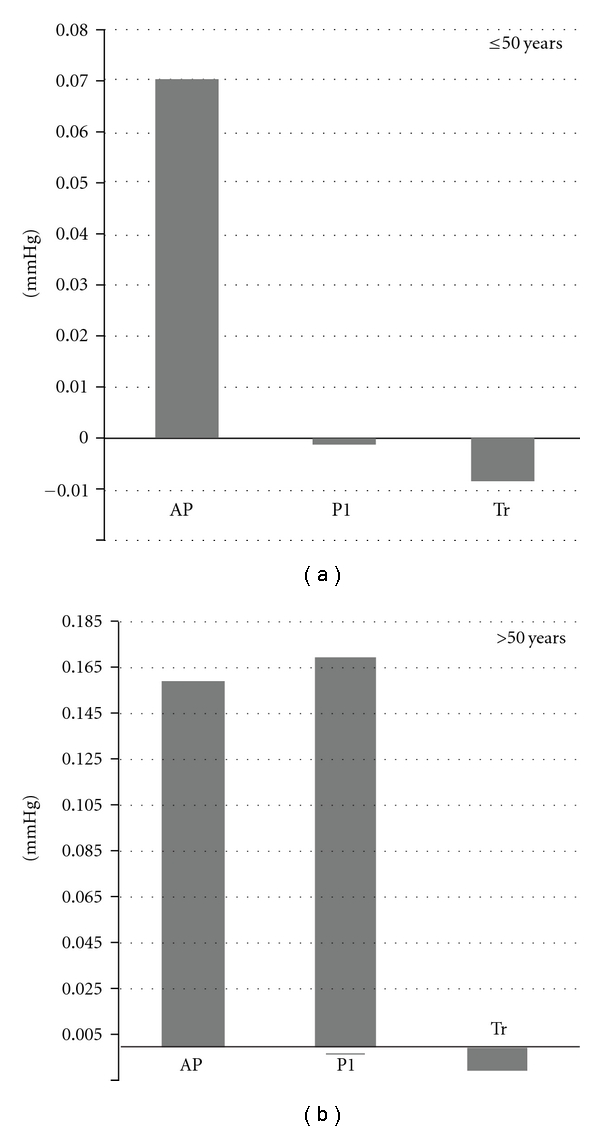
Per year contribution on augmented pressure (AP), incident pressure (P1), and round trip travel time younger and older subjects.

Among the significant mediators, the yearly contribution of AP to the CSP before age 50 was a standardized coefficient of 0.07/year. In those above 50 years old, AP and P1 contribute similarly, with factors of 0.16/year and 0.17/year, respectively, suggesting that AP and P1 are of similar importance. The contribution of the Tr was only significant in the older group at a factor of −0.01/year.  Figure 2 demonstrates the contribution of the mediators to CSBP in the older and younger age groups.

**Figure 3:**
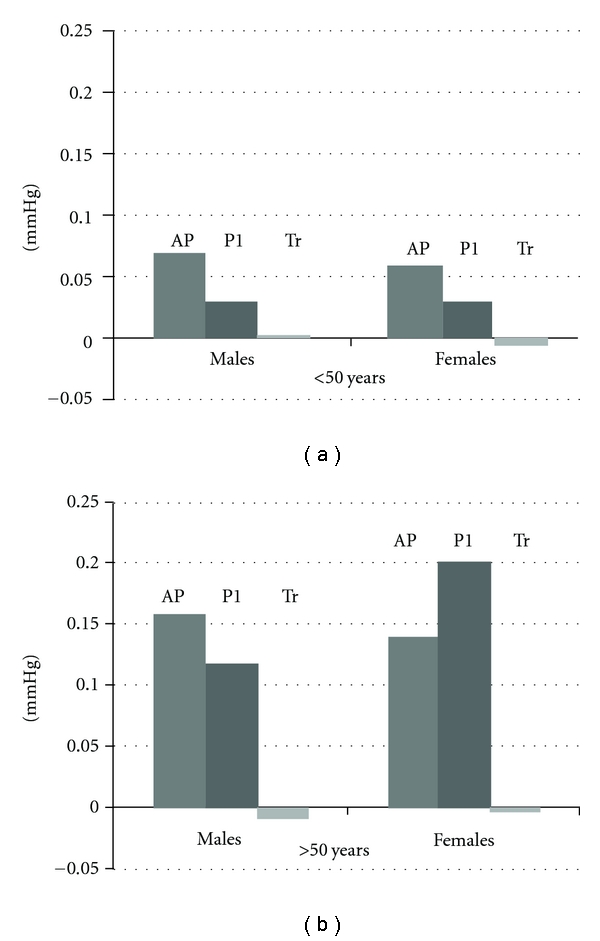
Gender-wise per year contribution of augmented pressure (AP), incident pressure (P1), and round trip travel time (Tr) to the aortic systolic pressure before and after 50 years.

**Figure 4:**
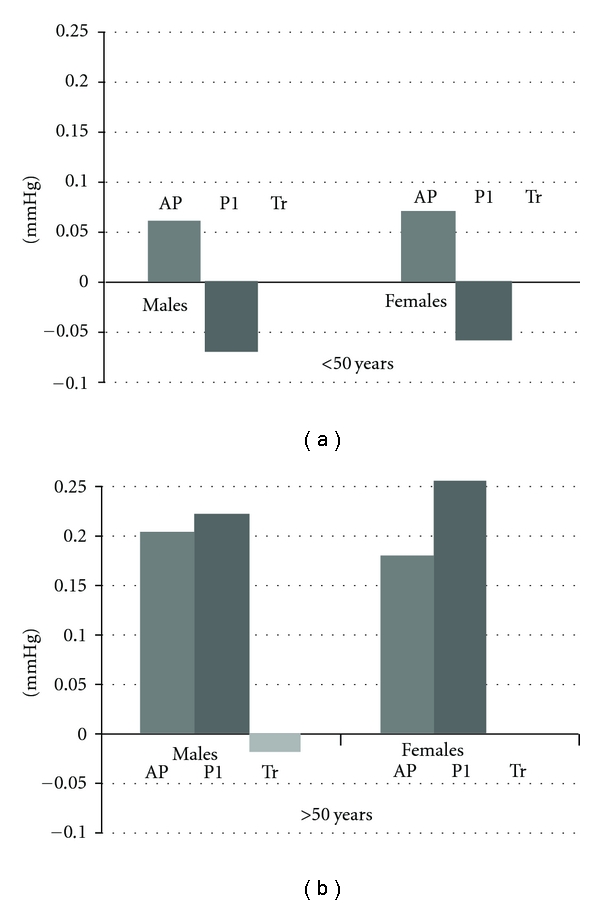
Gender-wise contribution of augmented pressure (AP), incident pressure (P1), and round trip travel time (Tr) to aortic pulse pressure before and after 50 years.

When analyzed by gender, among male subjects ≤50, CSP was mediated by AP, but not P1 or Tr (B = 0.07, 95% CI = 0.03–0.15, *P* < 0.05; B = −0.03, 95% CI = −0.09–0.05, *P* = NS; B = −0.0005, 95% CI = −0.07–0.16, *P* = NS, resp.). Among females ≤50 years, the CSP was mediated by AP, but not P1 or Tr (B = 0.06, 95% CI = 0.20–0.14, *P* < 0.05; B = −0.03, 95% CI = −0.09–0.2, *P* < NS; B = −0.008, 95% CI = −0.02– −0.0006, *P* < 0.05, resp.) (Figure 3). Among male subjects >50 years, CSP was mediated by AP, P1, and Tr (B = 0.16, 95% CI = 0.09–0.30, *P* < 0.05; B = −0.12, 95% CI = 0.03–0.20, *P* = <0.05 and B = −0.01, 95% CI = −0.0007– −0.08, *P* ≤ 0.05, resp.). Among females >50 years, the CSBP was mediated by AP, P1 and Tr (B = 0.14, 95% CI = 0.08–0.21, *P* < 0.05; B = −0.20, 95% CI = 0.14–0.28, *P* < NS; B = −0.004, 95% CI = −0.0001–0.01, *P* < 0.05, resp.) (Figure 3). When CPP was analyzed by gender and age group, the same mediators were found as for CSP (Figure 4).

When mediation analysis was performed with the inclusion of antihypertensive medication classes (beta blockers, ACE/ARB, and calcium channel blocking agents) as covariates, results were similar indicating that these medications had no effect on age and central pressure mediation by AP, P1, or Tr.

## 5. Discussion

These results suggest that in AA age ≤50 the effect of age on central pressures is primarily driven by AP alone, while in those >50 the contribution of age towards the formation of central pressures is equally mediated by AP and P1, with a small contribution from Tr. Similar results were found for both genders. The common medication classes of bêta blockers, angiotensin-converting enzymes inhibitors and angiotensin receptor blockers, and calcium channel blockers did not substantially alter out results. The mechanism of increasing central BP with age is an area of continued interest and discussion. Higher CSP and PP are markers and potential mediators of cardiovascular disease and have been found to be predictive of cardiovascular events [20–22]. There have been opposing viewpoints with regard to the relative contributions of the incident and reflected waves to CSP and PP [3]. Greater aortic impedance may be a predominant factor, especially in older subjects [9, 23]. The present study helps to clarify these relations among AA, a group of patients particularly vulnerable to hypertensive complications. The present study findings are similar to that of Namasivayam et al. [11], who in a healthy cohort found wave reflection to be important throughout life, while aortic impedance was significant in older subjects. They chose a partition age of 60 years for their analysis, based on physiologic considerations. A strength of our study is that we statistically determined 50 years to be the optimal partition based on our finding that only above this age did P1 and age correlate. This may be population dependent, since AAs have been suggested to have increased aortic stiffness as compared to whites. Regardless of the exact age, the present study underscores the facts that the reflected arterial wave continues to contribute to central aortic BP later in life. The contribution of the reflected wave was emphasized by the work of Murgo et al. [4], who found central BP augmentation related to waveform reflection and characterized waveforms in older and younger subjects. These findings have been confirmed by subsequent studies [24]. In addition, aortic stiffness and characteristic impedance may also contribute to a higher CSP, and it has been suggested that it is the primary mechanism. In a healthy middle aged to older cohort from the Framingham study, Mitchell et al. found an age-related increase in aortic PWV and the forward pressure wave, with little change or decrease in the AI [9]. Another study of hypertensive subjects found higher PP related to increased aortic wall stiffness and smaller aortic diameter even when corrected for AI [23]. Multiple studies of predominantly Caucasian subjects have shown differing changes in AI, AP, and PWV with age. AI rises in a curvilinear manner only to level off in older age although AP continues to increase throughout life. PWV remains stable in youth but increases in older subjects [15]. The curvilinear nature and plateau noted for AI is likely due to the similar rise in both AP and PP with aging [25]. These patterns have raised concern about the utility of AI in risk stratification of older populations [9].

In younger individuals, AI rises steeply with age whereas aortic PWV does not [14]. This is consistent with the idea that the rise in aortic pressures is due to an increase in the magnitude of wave reflection rather than increased aortic stiffness. In older individuals, AI changes little, however, aortic PWV increases, suggesting that the rise in aortic pressures is driven by both the earlier return of the reflected wave and increasing aortic stiffness and not only changes in the magnitude of wave reflection alone [15]. The magnitude of reflected wave is in part dependent upon the magnitude of incident wave, and, therefore, AP continues to rise in older individuals due to increasing P1. In our study, Tr mediated CSP to a lesser extent and only in the older group.

The aorta functions as a conduit to transport blood from the heart to the periphery and as a protective cushion to lessen pulsatile flow to the end organs. With advancing age, the arteries progressively stiffen. This phenomenon is more common in the large elastic arteries compared to the more muscular peripheral arteries and results in the progressive loss of vascular elasticity, decreased cushioning effect and worsening microvascular function and end organ damage [26, 27]. There is an increase in PP due to an increase in systolic pressure and a decrease in diastolic pressure. The two mechanisms that underlie increased PP appear to be a higher P1 generated by the ejection of blood from the left ventricle into a stiffened aorta, and an increase in AP due to higher P1 and a shorter Tr allowing the reflected wave to return in late systole and further augment systolic pressure [28]. Our results are consistent with these mechanisms, which result in higher central systolic and pulse pressures and are closely related to CV outcomes [2]. Left ventricular function is also adversely affected, by decreased diastolic coronary perfusion, increased left ventricular hypertrophy, and increased left ventricular workload and wasted pressure energy [29].

Our data is subject to the limitations of a cross-sectional study which restricts our ability to determine causal relationships of age and pressures. Longitudinal studies are lacking. We used patient interview and when feasible chart review to obtain clinical characteristics and history, which although standard is imperfect and may lead to error in data collection. Despite these limitations, however, our population of AA is the largest group to be studied for the age-related waveform components of central blood pressure. We used both multivariate and mediation analysis controlling for covariates to help sort out these relationships.

## 6. Conclusion

In summary, this study helps to clarify the physiology of increasing central blood pressure with age. The findings in our AA cohort are similar to a previously studied mostly Caucasian normal population [11]. CSP and PP are in part mediated by wave reflection throughout life, while increasing aortic impedance is important at an older age. Treatments aimed at decreasing wave reflection, such as vasodilators, may be more efficacious in the younger hypertensive population. Therapies to lower aortic stiffness, if and when available, would likely be of greater benefit in the older age group.

## Figures and Tables

**Table 1:** Clinical and hemodynamic characteristics of the study population.

	≤50 years (*n* = 289)	>50 years (*n* = 611)	
Variable	Mean ± SD	Mean ± SD	*P* value
Age (years)	38 ± 9	67 ± 10	<0.001
Males (%)	41	36	0.15
Weight (kg)	81.8 ± 21	79 ± 18	0.07
Height (m)	1.69 ± 0.10	1.67 ± 0.10	0.004
SBP (mmHg)	125 ± 21	139 ± 22.8	<0.001
DBP (mmHg)	78 ± 14	79 ± 13	0.201
PP (mmHg)	47 ± 13	60 ± 19	<0.001
MAP (mmHg)	93 ± 15	99 ± 14	<0.001
CSBP (mmHg)	112 ± 20	127 ± 22	<0.001
CDP (mmHg)	79 ± 14	81 ± 12	0.15
CPP (mmHg)	33 ± 11	47 ± 17	<0.001
CMAP (mmHg)	90 ± 15	96 ± 1⁄4	<0.001
HR (beats/min)	74 ± 14	69 ± 12	<0.001
ED (ms)	303 ± 37	308 ± 35	0.07
AP (mmHg)	6 ± 7	14 ± 9	<0.001
P1 (mmHg)	27 ± 7	33 ± 12	<0.001
Tr (ms)	144 ± 17	135 ± 15	<0.001
AI75 (%)	16 ± 14	25 ± 11	<0.001
AI (%)	16 ± 16	28 ± 12	<0.001
PWV (m/sec)	8.7 ± 1.6	9.2 ± 1.9	0.001
PPA	1.45 ± 0.2	1.30 ± 0.2	<0.001
Brachial-central SBP (mmHg)	13 ± 6	12 ± 7	0.028
Brachial-central PP (mmHg)	14 ± 6	12 ± 6	0.011
ΔTr (ms)	159 ± 37	174 ± 35	<0.001
HTN (%)	35	75	<0.001
Chol (%)	16	47	<0.001
DM (%)	13	38	<0.001
CAD (%)	8	21	<0.001

Abbreviations: SBP: systolic blood pressure, DBP: diastolic blood pressure, PP: pulse pressure, MAP: mean arterial pressure, HR: heart rate, ED: ejection duration, AP: augmented pressure, P1: incident pressure, Tr: round trip travel time, AI: augmentation index, AI@75: heart rate-corrected augmentation index, PWV: pulse wave velocity, PPA: pulse pressure amplification, ΔTr: reflected wave systolic duration, HTN: hypertension, Chol: hypercholesterolemia, DM: diabetes mellitus, CAD: coronary artery disease, kg: kilograms, mmHg: millimeters of mercury, ms: milliseconds, m: meters, m/sec: meters per second.
